# [Retracted] Limb remote ischemic post‑conditioning reduces injury and improves long‑term behavioral recovery in rats following subarachnoid hemorrhage: Possible involvement of the autophagic process

**DOI:** 10.3892/mmr.2024.13419

**Published:** 2024-12-17

**Authors:** Xiang Hu, Tao Lv, Shao-Feng Yang, Xiao-Hua Zhang, Yi-Feng Miao

Mol Med Rep 17: 21–30, 2018; DOI: 10.3892/mmr.2017.7858

Following the publication of the above paper, a concerned reader drew to the attention of the Editorial Office that the ‘Sham’ brain image featured in Fig. 1B on p. 23 was strikingly similar to an image that was published subsequently in the journal *Scientific Reports*, whereas the control TUNEL assay data shown in Fig. 4A on p. 25 were similarly strikingly similar to data shown in a paper published previously in the journal *Mediators of Inflammation*, even though the overall experiments portrayed in the other journals were different.

As the three affected articles did hold at least one author in common, we asked the authors to provide an explanation to account for the sharing of these data among these papers, but no reply was forthcoming from them; therefore, in the absence of a reply from these authors, the Editor of *Molecular Medicine Reports* has decided that this paper should be retracted from the Journal on account of a lack of confidence in the presented data. The Editor apologizes to the readership for any inconvenience caused.

